# Chronic noncommunicable diseases and their associated factors in community health workers

**DOI:** 10.47626/1679-4435-2021-921

**Published:** 2024-02-16

**Authors:** Larissa Santos Amorim Dias, Leonardo Evangelista Rezende, Luca Eleutério Salerno Del-Menezzi, Pedro Antônio Gusmão de Souza, Wanessa Casteluber Lopes, Lucineia Pinho

**Affiliations:** 1 Faculdade de Medicina, Centro Universitário FIPMoc, Montes Claros, MG, Brazil; 2 Programa de Pós-Graduação em Saúde, Sociedade e Ambiente, Universidade Federal dos Vales do Jequitinhonha e Mucuri, Diamantina, MG, Brazil; 3 Programa de Pós-Graduação de Cuidado Primário em Saúde, Unimontes, Montes Claros, MG, Brazil

**Keywords:** chronic, non-communicable diseases, community health agent, family health strategy, risk factors, doenças crônicas não transmissíveis, agente comunitário de saúde, estratégia saúde da família, fatores de risco

## Abstract

**Objectives:**

To analyze the epidemiological profile of chronic noncommunicable diseases among community health workers and associated factors.

**Methods:**

Cross-sectional study of community health workers in the city of Montes Claros, Minas Gerais, Brazil. Data were collected through a questionnaire designed to characterize demographic and socioeconomic profile, employment profile, self-reported presence of chronic noncommunicable diseases, and lifestyle habits. The variables were compared between participants with and those without chronic noncommunicable diseases, with Pearsons chi-square test used to define statistically significant differences between them.

**Results:**

674 community health workers participated in the study, 43.32% of whom self-reported the presence of at least one chronic noncommunicable disease; chronic respiratory diseases and hypertension were the most prevalent, especially in the age group > 34 years, those with > 10 years' experience as community health workers, overweight or obese participants, sedentary participants, and those employed as a civil servant or service provider.

**Conclusions:**

Our results show that community health workers have a prevalence of chronic noncommunicable diseases and risk factors thereof similar to that found in the general Brazilian population.

## Abstract (pt)

**Objetivos:**

Analisar o perfil epidemiológico das doenças crônicas não transmissíveis em agentes comunitários de saúde e fatores associados.

**Métodos:**

Tratou-se de estudo transversal, cuja população-alvo foram agentes comunitários de saúde no município de Montes Claros, no estado de Minas Gerais. A coleta dos dados foi realizada por meio de um questionário de pesquisa caracterizando o perfil demográfico e socioeconómico dos agentes comunitários de saúde, o perfil laboral, a presença de doenças crônicas não transmissíveis autorreferidas e os hábitos de vida. As variáveis foram avaliadas comparativamente entre não portadores e portadores de doenças crônicas, utilizando-se o teste do qui-quadrado de Pearson para definir diferenças estatisticamente significativas.

**Resultados:**

Participaram 674 agentes comunitários de saúde, e aproximadamente 43,32% dos participantes autorreferiram a presença de alguma doença crônica, sendo as doenças respiratórias crônicas e hipertensão arterial sistêmica as mais prevalentes, principalmente na faixa etária > 34 anos, tempo de trabalho como agentes comunitários de saúde > 10 anos, indivíduos com sobrepeso ou obesidade, sedentários e com o vínculo empregatício como concursado ou prestador de serviço.

**Conclusões:**

Os resultados mostraram que os agentes comunitários de saúde apresentam prevalência de doenças crônicas não transmissíveis e associações com fatores de risco semelhantes às encontradas na população brasileira.

## INTRODUCTION

Chronic noncommunicable diseases (NCDs) currently represent the leading global public health issue, accounting for 70% of all deaths worldwide.^1,2^ In Brazil, this group - composed of cardiovascular disease (CVD), chronic respiratory disease (CRD), malignancies, and diabetes mellitus (DM) - causes more than half of all deaths each year in the population aged 30 to 69, resulting in a massive economic impact on public health and social security^3,4^

These diseases have a multifactorial etiology, i.e., several factors have been identified that increase the risk of their development, all attributed jointly or individually to lifestyle (such as the way one lives, works, and ages). These factors include obesity (especially in women), physical inactivity, unhealthy dietary habits, harmful alcohol intake, smoking, and stress.^4^

Health care workers are exposed to many of these risk factors, including community health workers (CHWs), who, in Brazil, work within the framework of the Family Health Strategy (Estratégia de Saúde da Família, ESF). Part of the role of CHWs is to establish a channel for communication between health services and the community by carrying out interventions essentially designed to prevent disease, as well as promote activities that educate families about diseases and how to avoid ill health.^5^

Considering the roles and responsibilities of health care workers, one would expect that this group would have greater knowledge of health-related behaviors and their long-term consequences. Specifically, the behaviors of health care workers can affect patients' attitudes and encourage them to make lifestyle changes.^6^ However, their working hours and conditions, compounded by a knowledge barrier and issues with living habits, influence the emergence and progression of NCDs, posing an obstacle to attempts at adopting a healthy lifestyle.^2,5^

The objective of the study was to analyze the epidemiological profile of NCDs in CHWs and its association with socioeconomic, demographic, and work-related factors and lifestyle habits.

## METHODS

This was a cross-sectional study carried out in 2018 in the municipality of Montes Claros, located in the north region of the Brazilian state of Minas Gerais. Montes Claros has a population of 413,287 and is the main urban hub in its region. It is seat of one of the states health macro-regions^7^ and receives referrals from elsewhere in the state within the publicly funded health system.

The target population of the study comprised 797 CHWs distributed across 135 Family Health Strategy teams.^8^ All CHWs working within the municipality were invited to take part in the study. The following eligibility criteria were applied: 1) currently work in the role of CHW; 2) agree to participate in the study; and 3) complete the study questionnaire. Individuals who met the following criteria were excluded: 1) were absent from work or on leave, 2) were pregnant during data collection, or 3) answered the questionnaire incorrectly.

Data collection was carried out from March through December 2018 by a trained team within each ESF, under the supervision of the principal investigators. The study questionnaire was drawn from a preexisting instrument used in the Surveillance System of Risk and Protective Factors for Chronic Diseases by Telephone Survey for adults in Brazil (Vigitel),^9^ and sought to characterize:

**a)** the demographic and socioeconomic profile of CHWs. This section contained items on: sex (male or female), age (up to 34 years or 34 years and older), education (primary or secondary/higher), self-reported skin color/ethnicity (white or nonwhite), and marital status (has a partner or does not have a partner);
**b)** a self-reported occupational profile: years of service (up to 10 years or 10 years and over), weekly working hours (24 hours or 40 hours), employment relationship (contractor/employee or civil servant/service provider);
**c)** presence of self-reported NCDs, which included: malignancies, hypertension (HTN), DM, CVD (coronary artery disease and/or angina pectoris, myocardial infarction and/or coronary thrombosis, heart failure, other cardiovascular disease), chronic respiratory disease (chronic bronchitis, chronic sinusitis, asthma, emphysema, other);
**d)** risk factors: sedentary lifestyle (yes or no), body mass index (BMl) (less than or equal to 24.9 or greater than or equal to 25) calculated from directly measured weight and height, harmful alcohol intake (yes or no; defined as intake of more than seven standard drinks per week^10^), and current smoking (yes or no; individuals who had never smoked or had not smoked for more than 5 years were considered nonsmokers).

The collected data were categorized, processed, and analyzed in the SPSS* software environment. Initially, the demographic, socioeconomic, work-related characteristics and risk factors of the groups were evaluated descriptively. Pearsons chi-square test was used to compare behaviors and risk factors between participants with and without NCDs and ascertain whether statistically significant differences existed between these two groups. A logistic regression model was used to calculate odds ratios (ORs) as estimates of the magnitude of associations, considering a significance level of 5% (p < 0.05).

This study was approved by the Research Ethics Committee of Universidade Estadual de Montes Claros (opinion number 2,425,756) and was conducted in accordance with the ethical principles set forth in Brazilian National Health Council Resolution 466/2012.

## RESULTS

Of the 797 CHWs in the municipality of Montes Claros, 123 (15.3%) were excluded from the present study for various reasons (reassigned to another role; pregnant or on maternity leave at the time of data collection; less than 1 year experience; on medical leave; inappropriate questionnaire completion). Therefore, the sample comprised 674 CHWs. Most were female (83.8%) and over 34 years of age (57.1%). Regarding the main work-related variables and lifestyle habits, most participants declared that they had been working in the Family Health Strategy for 10 years or less (73%), had a weekly workload of 40 hours (93.8%), BMI ≥ 25 kg/m2 (61.7%), did not consume an unhealthy diet (59.6%), were not sedentary (73.9%), and did not engage in harmful alcohol intake (93.8%) or smoking (93.3%) (Table 1).

**Table 1:** Prevalence of concurrent chronic noncommunicable diseases and association with sociodemographic, work-related, and lifestyle variables

Sociodemographic characteristics	n	%	No concurrence (0 or 1 disease) (n = 627)			Concurrence (2 to 5 diseases) (n = 47)			
			n	%	95%CI	n	%	95%CI	p-value*
Sex									0.511
Male	109	16.2	103	94.5	(88.5-975)	6	5.5	(2.6-11.5)	
Female	565	83.8	524	92.7	(90.3-94.6)	41	7.3	(5.4-9.7)	
Age (years)									0.001
≤34	289	42.9	280	96.9	(94.2-98.4)	9	3.1	(1.7-5.8)	
>34	385	57.1	347	90.1	(86.7-92.7)	38	9.9	(73-13.3)	
Marital status									0.558
No partner	271	40.2	254	93.7	(90.2-96.1)	17	6.3	(4.0-9.8)	
Has partner	403	59.8	373	92.6	(89.6-94.7)	30	7.4	(5.3-10.4)	
Educational attainment									0.916
Primary	22	3.3	20	90.9	(72.2-975)	2	9.1	(2.5-278)	
Secondary	487	72.3	453	93.0	(90.4-95.0)	34	7.0	(5.0-9.6)	
Higher	165	24.5	154	93.3	(88.5-96.2)	11	6.7	(3.8-11.5)	
Skin color									0.268
White	87	12.9	78	89.7	(81.5-94.5)	9	10.3	(5.5-18.5)	
Black	97	14.4	88	90.7	(83.3-95.0)	9	9.3	(5.0-16.7)	
Mixed/Brown	476	70.6	447	93.9	(91.4-95.7)	29	6.1	(4.3-8.6)	
Other	14	2.1	14	100.0	(78.5-100.0)	0	0.0	(0.0-21.5)	
Time working as CHW (years)									0.013
≤10	492	73.0	465	94.5	(92.1-96.2)	27	5.5	(3.8-7.9)	
>10	182	27.0	162	89.0	(83.6-92.8)	20	11.0	(72-16.4)	
Overweight									0.001
No	263	39.2	257	97.7	(95.1- 98.9)	6	2.3	(1.1-4.9)	
Yes	408	60.8	367	90.0	(86.7-92.5)	41	10.0	(75-13.4)	
Diet									0.121
Healthy	272	40.4	248	91.2	(872-94.0)	24	8.8	(6.0-12.8)	
Unhealthy	402	59.6	379	94.3	(91.2-96.2)	23	5.7	(3.8-8.4)	
Smoker									0.259
No	629	93.3	587	93.3	(91.1-95.0)	42	6.7	(5.0-8.9)	
Yes	45	6.7	40	88.9	(76.5-95.2)	5	11.1	(4.8-23.5)	
Alcoholic?									0.055
No	632	93.8	591	93.5	(91.3-95.2)	41	6.5	(4.8-8.7)	
Yes	42	6.2	36	85.7	(72.2-93.3)	6	14.3	(6.7-278)	
Sedentary lifestyle									0.008
No	498	73.9	471	94.6	(92.2-96.2)	27	5.4	(3.8-7.8)	
Yes	176	26.1	156	88.6	(83.1-92.5)	20	11.4	(74-16.9)	
Weekly workload (hours)									0.561
24	42	6.2	40	95.2	(84.2-98.7)	2	4.8	(1.3-15.8)	
40	632	93.8	587	92.9	(906-94.7)	45	71	(5.4-9.4)	
Employment relationship: contractor									0.003
No	177	26.3	156	88.1	(82.5-92.1)	21	11.9	(79-17.5)	
Yes	497	73.7	471	94.8	(92.5-96.4)	26	5.2	(3.6-7.5)	

* Pearson's chi-square.

CHW = community health worker.

Approximately 43.32% of participants self-reported the presence of any NCD. The most prevalent were CRD (18.2%), HTN (14.8%), and CVD (6.1%), while DM (3.9%) and malignancy (0.3%) were the least prevalent. Among CRDs, the most common was chronic sinusitis; among CVDs, coronary heart disease and angina pectoris predominated (Table 2).

**Table 2:** Prevalence of chronic noncommunicable diseases in community health workers

Variable/self-reported chronic noncommunicable disease	n	%
Hypertension	100	14.8
Diabetes mellitus	26	3.9
Cancer	2	0.3
Cardiovascular disease	41	6.1
Chronic respiratory disease	123	18.2

Regarding the factors associated with NCDs in CHWs, the following variables were statistically significant on bivariate analysis: age > 34 years (p = 0.001), time working as a CHW > 10 years (p = 0.013); BMI classified as overweight or obese (p = 0.001), sedentary lifestyle (p = 0.008), and having an employment relationship as a civil servant or service provider (p = 0.003) (Table 1). In the multivariable model, the variables age (OR = 2.5; 95%CI 1.1-5.4; p = 0.027), BMI (OR = 3.7; 95%CI 1.5-9.1; p = 0.004), and sedentary lifestyle (OR = 2.3; 95% CI 1.2-4.3; p = 0.009) were significant.

## DISCUSSION

The present study found that approximately 43% of the queried population have NCDs. Melo et al.^11^ previously found an NCD prevalence of 56.7% among adults living in an urban area in northeastern Brazil. The country's household-based survey which covers approximately 64 000 households and is carried out by the Brazilian Institute of Geography and Statistics (Instituto Brasileiro de Geografia e Estatística - IBGE),^12^ found in 2013 that the prevalence of some NCDs in the overall population was up to 45%.

NCDs now constitute a serious problem for the national health system and are among the leading causes of death in the country^13^; they are also strongly associated with an increased number of outpatient visits, hospitalizations, and absences from work.^3^ Over the last few decades, the context of mortality and morbidity across Brazil has shifted markedly as a result of the epidemiological transition, with a decline in infectious diseases and an increase in NCDs, accidents, and violence as causes of illness and death; this phenomenon is also linked to a substantial demographic and nutritional transition.^2,4^

Among the conditions classified as NCDs for the purposes of this study, chronic respiratory diseases were self-reported by approximately one-fifth of respondents. This differs from a national study in which hypertension, followed by chronic back problems, appeared more frequently^14^ In a study carried out by Leal et al.,^15^ the self-reported prevalence of chronic respiratory disease was 3%. Another nationwide study found a substantial increase in other variables in the spectrum associated with conditions that are related to the increase in CRD rates, such as hypertension, DM, overweight, obesity, and harmful alcohol use.^9^

The prevalence of hypertension tends to follow national rates, at approximately 15%. In a field survey of workers employed at health care facilities in Campo Grande, state of Mato Grosso do Sul, HTN was reported by 25% of the sample, a finding that highlights the difficulty even health care professionals experience in keeping CVD risk factors under control, adopting a healthier lifestyle, and protecting themselves from this condition.^16^

There is a well-known relationship between age and prevalence of HTN. In an analysis of Vigitel findings from 2006 to 2014, there was an 18% increase in the rate of HTN cases from the 18-to-29 age range to the 30-to-59 age range. Within this context, other risk factors contribute directly to the increase in the number of cases of HTN, such as being overweight (BMI > 25) - this was well demonstrated by the Vigitel study, in which 52% of the sample was overweight - as well as obesity (BMI > 30), which was predominant in female participants and those aged 35 to 64 years.

Furthermore, the importance of dietary factors, such as salt intake and consumption of alcoholic beverages, and - above all - a sedentary lifestyle cannot be overstated.^17^ Among sociodemographic characteristics, age over 34 years is associated with greater susceptibility to NCDs, the result of a greater life expectancy due to reduced mortality from other diseases as well as plans and goals to reduce premature mortality in patients with these conditions.^18^

In the present study, the prevalence of NCDs in overweight or obese participants was 87.23%. An association study of NCDs and their risk factors carried out by Rocha-Brischiliar et al.^19^ found that the risk of developing these diseases was 3.55 times greater among those with a BMI ≥ 25 kg/m². Obesity is one of the main risk factors for emergence of NCDs. It is demonstrably associated with greater cardiovascular risk and morbidity, diseases such as hypertension, and even some types of cancer.^20^ Furthermore, the obesity epidemic of recent years has been associated with a population-wide increase in the prevalence of DM, causing risk factors to accrue and posing challenges to interventionist policies,^21^ demonstrating the impact of this risk factor on the emergence and, consequently, prevalence of NCDs.

We also found that 61.72% of the CHWs had a BMI equal to or greater than 25, even though they work in the health sector and theoretically would have greater knowledge about overweight, obesity, and their risks. When CHWs are careless with their own health, this can compromise their power of persuasion over the population they serve - i.e., when members of the community realize that health educators do not "practice what they preach" they may no longer be compelled to improve their health.^22^

In our sample, the number of years working as a CHW was associated with increasing prevalence of NCDs. This may be at least partly explained by the fact that the work carried out by health care providers is highly taxing from a psychological standpoint, consequently generating high levels of stress.^23^ Studies have shown that stress plays an important role in the etiology of many diseases and is detrimental to health.^24^ The scope of CHW practice in Brazil encompasses the whole life course of families and priority health conditions; they follow people throughout the cycle of birth, life, illness, and death.^25^ While attempting to implement the interventions defined by Unified Health System (SUS) protocols and managers, they experience the reality of life in impoverished communities like few others.^26^ Furthermore, when they live in the community they serve, CHWs may be overwhelmed by unrecognized off-hours work: due to their proximity, they are often sought out by community members at unconventional times, including overnight, on weekends and holidays.^25,26^

NCDs are one of the leading public health issues worldwide, accounting for 68% of all global deaths and burdening the working-age population with morbidity. In recent decades, the incidence of these diseases has increased as a result of the rapid demographic transition of Brazil, in addition to rising rates of obesity and increased prevalence of various risk factors such as unhealthy eating, physical inactivity, alcohol intake, and smoking. In addition to its repercussions at the individual level, the NCD epidemic has serious consequences for society by jeopardizing quality of life and overloading health systems. In one study, the presence of at least one NCD was associated with a 14.8% increase in health services utilization by the Brazilian adult population and a 1.7-fold increase in the number of hospitalizations; it is estimated that the socioeconomic costs associated with these diseases in low and medium-income countries between the years 2011 and 2025 will amount to US$ 7 trillion. Addressing NCDs thus becomes a necessary condition for the development of these countries in the 21st century^2,3^

Having identified this need, the Brazilian Ministry of Health has developed several public policies to monitor and prevent these conditions, such as conducting the Vigitel survey in 2006 and dispensing antihypertensive and antidiabetic medications free of charge. In 2011, it devised the *Strategic plan of action to address NCDs in Brazil 2011-2022,* which aims to promote the development and implementation of effective, integrated, sustainable, evidence-based public policies for the prevention and control of NCDs and their risk factors, thus preparing the country to face and stop the rise of NCDs in the next decade, based on surveillance, information, monitoring and evaluation, health promotion, and comprehensive care.^27^

One limitation of the present study is the use of data self-reported by CHWs themselves. Self-reporting may underestimate the actual prevalence of detrimental health habits and, therefore, may represent a source of bias in the interpretation of survey results. The cross-sectional design is an additional limitation, as it precludes any causal inference.

## CONCLUSIONS

The results showed that CHWs experience a prevalence of NCDs equivalent to that found in the general Brazilian population. In this group, NCDs were associated with sociodemographic, work-related, and lifestyle characteristics, corroborating the findings of several previous studies and adding to the reliability of the data obtained. This study highlights the need for greater attention to CHWs in NCD prevention and control programs, as CHWs are a vital link between health teams and the community and must be committed to improving health indicators.
